# Potential induction of the relative mRNA expression levels of CYP450 by Zhicaowu-Hezi (Aconiti kusnezoffii radix preparata and *Terminalia chebula* Retz.)

**DOI:** 10.3389/fphar.2025.1573739

**Published:** 2025-07-14

**Authors:** Junxuan Zhu, Ming An, Weiting Wang, Jingjing Guo, Mengting Chen, Longlong Fang, Cen Wang, Dong Zhang, Guodong Wu

**Affiliations:** ^1^ School of Pharmacy, Baotou Medical College, Baotou, China; ^2^ School of Basic Medical Sciences and Forensic Medicine, Baotou Medical College, Baotou, China

**Keywords:** Aconiti kusnezoffii Radix Preparata, *Terminalia chebula* Retz., cocktail probe drug method, CYP450, pharmacokinetics

## Abstract

**Background:**

Processed Aconiti Kusnezoffii Radix (Aconiti kusnezoffii Radix Preparata, Zhicaowu) and *Terminalia chebula* Retz. (Hezi) are a classic herb pair in Mongolian medicine, where Hezi mitigates Zhicaowu’s hepatotoxicity. Despite extensive studies on their detoxification effects, the role of cytochrome P450 (CYP450) modulation remains unclear.

**Aim:**

This study aimed to systematically evaluate the regulatory effects of Zhicaowu and Hezi combination on both enzymatic activity and mRNA expression of CYP450 isoforms (CYP1a2, CYP2b1, CYP2c11, CYP2c13, CYP2d2, CYP2e1, and CYP3a1), and to explore their correlation with hepatoprotective effect.

**Methods:**

The effects of Zhicaowu-Hezi formulation on CYP450 enzymes were systematically evaluated through integrated *in vivo* and *in vitro* approaches. Rats received 14-day oral administrations of either Zhicaowu, Hezi, or their combinations (1:1, 1:3, 3:1 ratios), followed by comprehensive assessment using: (1) cocktail probe drug assays monitoring seven CYP450 isoforms (CYP1a2, 2b1, 2c11, 2c13, 2d2, 2e1, 3a1) with HPLC quantification methods for substrate detection, (2) RT-qPCR analysis of hepatic CYP450 mRNA expression, and (3) parallel *in vitro* studies employing rat liver microsomes to verify enzyme activity changes. These pharmacological evaluations were correlated with histopathological and biochemical indices to establish mechanistic relationships between CYP450 modulation and hepatotoxicity attenuation.

**Results:**

Pathological and biochemical analyses confirmed Hezi’s hepatoprotective effects against Zhicaowu-induced toxicity, with the 1:3 Zhicaowu-Hezi combination showing optimal efficacy. *In vivo* pharmacokinetic studies revealed that Zhicaowu significantly inhibited CYP1a2, CYP2d2, CYP3a1, and CYP2c11 activities, as demonstrated by marked increases in the AUC_(0-t)_, AUC_(0-∞)_), and C_max_ values of their respective probe substrates (theophylline, metoprolol, testosterone, and diclofenac), along with significantly prolonged t_1/2_ z and reduced CLz/F. It is worth noting that the combined use of Hezi effectively reversed these changes by inducing CYP450, causing significant alterations in the pharmacokinetic parameters of these four substrates. Complementary *in vitro* studies using liver microsomes consistently showed that Hezi treatment significantly enhanced the metabolic clearance of these four substrates. At the molecular level, RT-qPCR analysis demonstrated that Zhicaowu significantly suppressed hepatic CYP1a2, CYP2d2, CYP3a1, and CYP2c11 mRNA expression, while Hezi co-treatment restored their expression to normal or elevated levels.

**Conclusion:**

Hezi dose-dependently induced CYP450 enzyme activity, reversing Zhicaowu’s inhibition of CYP1a2/2d2/3a1/2c11 and markedly improving liver function and histopathology. These results elucidate the scientific basis for toxicity reduction in Zhicaowu-Hezi herb pair through metabolic enzyme regulation, supporting its traditional use. Future studies will focus on toxic alkaloid (e.g., aconitine) pharmacokinetics and their transcriptional regulatory pathways.

## 1 Introduction

Mongolian medicine is one of the four major ethnic forms of medicine in China, embodying a distinctive medical theory and therapeutic approach developed by the Mongolian people through centuries of practical experience and adaptation to disease.

Aconiti Kusnezoffii Radix (Chinese: Caowu) consists of the processed dried tuberous roots of *Aconitum kusnezoffii* Reichb., a species belonging to the buttercup family (Ranunculaceae). Caowu is often used in Mongolian medicine to eliminate mucus and relieve pain (Luo, 2006). Modern pharmacological studies have shown that Caowu has significant pharmacological effects such as analgesia, anti-inflammatory, anti-tumor, cardiotonic, antioxidant, and immune enhancement. (Jin et al., 2023; Li et al., 2013; Gao et al., 2022; Li et al., 2021). However, its active components, alkaloids, are highly toxic, and even minimal amounts can cause severe damage to vital organs, including the heart, liver, nervous system, and kidneys, among which cardiac toxicity is the most common (Yan et al., 2017; Gao et al., 2022; Wang et al., 2018). Therefore, in clinical applications, only the processed Caowu (Aconiti kusnezoffii Radix Preparata, Zhicaowu), the detoxifying agent of Caowu - is used. Compared with the crude preparation, the toxicity of Zhicaowu is significantly reduced.

Hezi is the dried mature fruit of the deciduous tree *Terminalia chebula* Retz. var. tomentella Kurt. or the downy *T. chebula* Retz. var. tomenteila Kurt. (Luo, 2006). Hezi is renowned for its detoxification, antibacterial, anti-inflammatory, and antioxidant effects and is often referred to as the king of Mongolian medicine (Pellati et al., 2013; Doye et al., 2023; El-Shamarka et al., 2024; Nigam et al., 2020).

Hezi is frequently integrated into Caowu (or Zhicaowu)-containing formulae to mitigate their toxicity. The use of Hezi soup for detoxifying Caowu (or Zhicaowu) poisoning has been a long-standing practice. The 19th-century “Canon of Mongolian Medicines” emphasized the necessity of incorporating Hezi in Caowu-containing formulations. The mechanism by which Hezi, when paired with Caowu (or Zhicaowu), mitigates their toxicity has recently become a prominent area of research.

Hezi contains tannins that can bind to alkaloids, thereby slowing their release and elimination. This interaction is believed to reduce the toxicity of diester-type diterpene alkaloids, enabling Hezi to exert a detoxifying effect on Caowu (Wang et al., 2002). Aconitine is known to induce arrhythmia, while Hezi addition has been shown to ameliorate myocardial electrophysiological abnormalities, contributing to both detoxification and cardioprotection (Liang et al., 2017). In an experiment investigating the protective effects of Hezi extract against aconitine-induced cardiomyocyte damage in lactating rats, Hezi significantly reduced lactate dehydrogenase and creatine kinase leakage, decreased malondialdehyde production, and enhanced superoxide dismutase activity. These findings suggest that Hezi protects cardiomyocytes through cell membrane stabilization and antioxidant activity (Zhang et al., 2010; Erdemli et al., 2018). A metabolomic-based study on the protective effects of Hezi against Caowu-induced cardiotoxicity found that such cardiotoxicity may be linked to disruptions in energy, nucleic acid, and amino acid metabolism and that Hezi mitigates cardiotoxicity by modulating these metabolic pathways (Liu et al., 2021). In summary, extensive research has been conducted on the detoxification mechanisms of the Caowu–Hezi combination. However, research on the interaction between Zhicaowu and Hezi based on cytochrome P450 (CYP450) enzymes remains limited.

The CYP450 superfamily comprises some of the most important drug-metabolizing enzymes in the human body. As the primary phase I enzyme system, it is responsible for metabolizing a wide range of exogenous and endogenous substances, including herbal compounds. This superfamily consists of three major families, CYP1, CYP2, and CYP3, each containing subfamilies and numerous individual enzymes (Wang et al., 2022; Rendic, 2002). Specifically, approximately 75% of drug biotransformation in the human body is catalyzed by CYP450 enzymes, which facilitate the conversion of drugs into polar compounds through oxidation, reduction, and hydrolysis. These metabolites are subsequently excreted from the body (Lu et al., 2015; Guengerich, 2013; Fan et al., 2014). CYP450 enzymes can be readily inhibited or induced by exogenous substances, thereby altering drug metabolism. Such effect may lead to changes in drug plasma concentration, potentially affecting therapeutic efficacy or toxicity (Zhang et al., 2018). The liver serves as the primary site of drug metabolism, with major CYP450 isoforms involved in this process, including CYP1A2, CYP2D6, CYP2B6, CYP2C19, CYP2E1, CYP3A4, and CYP2C9. These enzymes are homologous to the corresponding rat isoforms: CYP1a2, CYP2d2, CYP2b1, CYP2c13, CYP2e1, CYP3a1, and CYP2c11 (Ingelman-Sundberg, 2004; Sun et al., 2017). As drugs can regulate CYP450 enzyme activity, this regulation may, in turn, influence the metabolism of other compounds in a combination therapy. Consequently, herbal medicine pairings are more prone to involve CYP450-mediated interactions. An increasing number of studies have explored detoxification mechanisms in drug combinations via CYP450 enzyme modulation. For example, when Lei Gong Teng was combined with licorice, the metabolic production of Lei Gong Teng methylin and lactone significantly increased. This effect was attributed to licorice-induced CYP450 enzymes, by which accelerated the metabolism of these toxic compounds, thereby mitigating Lei Gong Teng toxicity (Liu et al., 2010). Similarly, a study on licorice’s detoxification against *Epiphyllum* toxicity found that *Epiphyllum* inhibited CYP1A2 activity, whereas licorice induced CYP1A2 activity. When used in combination, the inductive effect of licorice on CYP450 enzymes likely accelerates the metabolism of *Epiphyllum*’s toxic constituents, thereby reducing its toxicity (Li et al., 2019). The primary toxic alkaloids of Zhicaowu—aconitine, hypaconitine, and neoaconitine—undergo key metabolic transformations such as demethylation, hydroxylation, and dehydrogenation. These toxic components are primarily metabolized by CYP1A2, CYP2D, CYP3A, and CYP2C enzymes. (Li et al., 2023; Miao et al., 2016; Tang et al., 2011; Ye et al., 2011; Wang et al., 2006). Previous studies have demonstrated that the aqueous extract of Hezi significantly inhibits CYP2C19 and 2E1 activities (Wu et al., 2020). Based on these findings, the present study therefore aims to investigate the herb-herb interaction between Zhicaowu and Hezi through CYP450-mediated pathways and explore their potential detoxification mechanisms.

We employed specific probe drugs for each CYP450 isoform: theophylline (CYP1a2), metoprolol (CYP2d2), bupropion (CYP2b1), omeprazole/mephenytoin (CYP2C13), chlorzoxazone (CYP2e1), testosterone (CYP3a1), and diclofenac (CYP2C11). Two optimized high-performance liquid chromatography (HPLC) methods were developed for simultaneous quantification of these seven probe drugs in both plasma and hepatic microsomal samples. To comprehensively evaluate the herb-herb interaction, we performed quantitative reverse transcription PCR (RT-qPCR) to assess hepatic CYP450 mRNA expression profiles in rats treated with Zhicaowu alone *versus* those treated with Zhicaowu-Hezi combination. By integrating histopathological examination and serum biochemical analysis, we adopted a multimodal approach to: (1) determine whether the CYP450 activity modulation mediated by Hezi, Zhicaowu, or their herb pair occurs via transcriptional regulation of the corresponding isoforms; and (2) establish potential correlations between these metabolic changes and the alleviation of Aconitum-induced hepatotoxicity. Our findings provide mechanistic insights into the detoxification effects of Zhicaowu-Hezi combination, while advancing the scientific basis for its clinical application in traditional medicine. This work also contributes to the growing understanding of pharmacodynamic interactions in herbal combination therapies.

## 2 Materials and methods

### 2.1 Plant materials and preparation

Aconiti Kusnezoffii Radix (Caowu) is the dried tuberous roots of *A. kusnezoffii* Reichb. (family Ranunculaceae). Aconiti kusnezoffii Radix Preparata (Zhicaowu) is the processed form of Aconiti Kusnezoffii Radix. Zhicaowu was purchased from Xinlin Aconite Pharmaceutical Co., Ltd. (Jiangyou, Sichuan, China; batch no. 200803) and Hezi was obtained from Ronghua Herbal Medicine Co., Ltd. (Anguo, Hebei, China; batch no. C550220601). Both materials were morphologically authenticated by Prof. Na Zhang (School of Pharmacy, Baotou Medical College).

The crude drugs were pulverized with a grinder and sieved through a 100-mesh sieve (150 μm nominal pore size). The resulting powders were stored in desiccators at room temperature until use.

### 2.2 Drug formulation

For animal administration, powders were freshly suspended in 0.3% (w/v) sodium carboxymethyl cellulose (CMC-Na, batch #20150912; Tianjin Kaitong Chemical Reagent Co., China) solution at specified w/w ratios (Zhicaowu:Hezi = 3:1, 1:1, 1:3). The suspensions were homogenized by vortex mixing (2000 RPM, 3 min) followed by ultrasonication (40 kHz, 5 min) to ensure uniformity. All suspensions were prepared and administered within 1 h to minimize sedimentation.

### 2.3 Other chemical reagents

Omeprazole and chlorzoxazone (purity >98%) were purchased from Meilun Biotechnology Co., Ltd. (Dalian, China). Theophylline, metoprolol, bupropion, testosterone, diclofenac, tinidazole (internal standard, IS), and NADPH (all purity >98%) were obtained from Efar Biotechnology Co., Ltd. (Chengdu, China). The reagents for the measurements of alanine aminotransferase (ALT), aspartate aminotransferase (AST), and alkaline phosphatase (ALP) were purchased from Gerace Biotechnology Co. TRNzol Universal Reagent, FastKing gDNA Dispelling RT SuperMix kit, and FasReal qPCR PreMix (SYBR Green) kits were procured from Tiangen Biochemical Science and Technology Co., Ltd. (Suzhou, China). Chromatography-pure acetonitrile was purchased from MREDA (United States), phosphoric acid from Tianjin Windship Chemical Reagent Technology Co., Ltd. (Tianjin, China), and ultrapure water was prepared using a GENPURE UV/UF-TOC ultrapure water apparatus (Thermo Fisher Scientific, United States). All the other reagents were of analytical grade.

### 2.4 Animals and experimental grouping

Six-week-old male Sprague–Dawley rats (SPF grade, 200 ± 20 g) were certified healthy (Spivey Biotech Ltd., Beijing, China). The license number for the rats was SCXK (Beijing) 2019–0010. The rats were housed in an animal facility of Baotou Medical College under a 12-h light-dark cycle, 50%–60% humidity, and 20°C–25°C temperature. All animals were acclimatized for 1 week before the experiments. The study was approved by the Institutional Animal Care and Use Committee (IACUC) of Baotou Medical College (approval no. 2022–96).

In the *in vivo* experiment, the rats were randomly divided into seven groups: a blank control group (Group C), Zhicaowu group (Group Z), Hezi group (Group H), Zhicaowu-Hezi 3:1 group (Group Z-H1), Zhicaowu-Hezi 1:1 group (Group Z-H2), Zhicaowu-Hezi 1:3 group (Group Z-H3), and phenobarbital sodium inducer group (Group P). Group C received 0.3% CMC-Na solution by gavage, while Groups Z, H, Z-H1, Z-H2, and Z-H3 were administered Zhicaowu (0.27 g/kg/d), Hezi (0.27 g/kg/d), and different proportional suspensions of Zhicaowu-Hezi in CMC-Na (with Zhicaowu maintained at 0.27 g/kg/d in all combination groups), respectively, via gavage for 14 days. Additionally, Group P was treated with intraperitoneal injections of phenobarbital sodium (0.05 g/kg/d) for 7 days. During the administration period, the rats were weighed at 3-day intervals, and the administered dose was adjusted according to the body weights. For the rat liver microsomes (RLM) study *in vitro*, rats were randomly divided into six groups following the same treatment protocol as the *in vivo* experiment, excluding Group P.

### 2.5 HPLC analysis conditions

The mixed samples were analyzed using an Ultimate 3000 HPLC system equipped with a diode array detector. The probe drug and IS were separated at 35°C using an Eclipse XDB-C18 column (4.6 mm × 250 mm, 5 μm; Agilent, United States). The mobile phase consisted of acetonitrile (A) and 0.1% aqueous phosphoric acid (B) with a gradient elution at a flow rate of 1 mL/min with an injection volume of 20 μL. The elution procedure was as follows: acetonitrile increased from 10% to 38% between 0 min and 15 min and from 38% to 70% between 16 min and 30 min, while it remained at 70% until 33 min.

The HPLC method was validated according to the requisite standards for biological sample analysis, including tests of specificity, precision, and accuracy (Scriba and Wätzig, 2018).

### 2.6 Effects of Zhicaowu–Hezi pairing on CYP450 activity in rats *in vivo*

The effect of the Zhicaowu–Hezi combination on CYP450 enzymes in rats was evaluated by examining the pharmacokinetics of theophylline (CYP1a2), metoprolol (CYP2d2), bupropion (CYP2b1), omeprazole (CYP2c13), chlorzoxazone (CYP2e1), testosterone (CYP3a1), and diclofenac (CYP2c11).

On Day 15, the rats in each group were administered a combination of seven cocktail probe drugs *via* gavage, and blood was collected from the posterior venous plexus of the eye at 0, 0.25, 0.5, 1, 1.5, 2, 4, 6, 8, 12, and 24 h after gavage. The collected blood was placed in sodium heparin-treated centrifuge tubes and centrifuged at 4°C for 10 min at 3,000 RPM. Precisely 100 μL of rat plasma samples were aspirated into 1.5 mL EP tubes, add 100 μL of acetonitrile solution containing dinidazole internal standard (39.65 μg/mL), vortexed for 30 s, sonicated for 10 min, and then centrifuged at 4°C for 5 min at 13,000 RPM. After 10 min of sedimentation, the supernatant was extracted through a 0.22-μm microporous membrane, 20 μL of which was injected into HPLC. The pharmacokinetic parameters of each probe drug were calculated using the non-atrial compartment model in Drug and Statistics software (DAS, 3.0; Shanghai, China).

### 2.7 Effect of Zhicaowu–Hezi pairing on CYP450 activity in RLM

The effect of the Zhicaowu–Hezi combination on CYP450 enzymes in RLM was evaluated by assessing the metabolic clearance rates of theophylline, metoprolol, bupropion, mephenytoin (CYP2c13), chlorzoxazone, testosterone, and diclofenac.

$$\text{Metabolic clearance rate }\left(\%\right)=\left[\left(\text{total drug concentration}-\text{residual drug concentration}\right)/\text{total drug concentration}\right]\times 100\%$$

#### 2.7.1 Preparation of RLM

On day 15, the rats were humanely euthanized, and liver tissues were excised for microsomal preparation. The calcium precipitation method was employed to prepare the RLM (Shi et al., 2020). Livers from each group were repeatedly washed with ice-cold phosphate buffered saline (PBS). Following excision, the livers displayed an earthy yellow coloration. Subsequently, they were blotted dry with filter paper, weighed, and homogenized by adding 4 mL of ice-cold PBS per gram of liver tissue. The mixture was homogenized in an ice bath. The liver homogenate was subjected to centrifugation at 10,000 RPM for 20 min at 4°C. The resulting supernatant was then combined with an ice-cold 88-mmol/L CaCl_2_ solution at a 1:1 ratio, and the mixture was homogenized in an ice bath for 5 min. The resulting solution was then centrifuged at 25,000 × *g* for 30 min at 4°C. The supernatant was discarded and the precipitate was repeatedly resuspended in PBS–20% glycerol buffer. The precipitate was then stored in a separate bottle at −80°C for backup. The protein concentration of the prepared RLM was determined using a bicinchoninic acid protein assay kit.

#### 2.7.2 RLM incubation experiments

The experimental reliability was evaluated using the metabolic clearance rates of seven enzyme-specific inhibitors, namely, α-naphthoflavone (CYP1a2), quinidine (CYP2d2), tiotropium (CYP2b1), ticlopidine (CYP2c13), 4-methylpyrazole (CYP2e1), ketoconazole (CYP3a1), and sulphamethoxazole (CYP2c11).

The experiment was divided into four distinct groups: an inactive control group, a blank control group, an administered group, and an inhibitor group. The inhibitor group was subdivided into seven groups (α-naphthoflavone, quinidine, tiotropium, ticlopidine, 4-methylpyrazole, ketoconazole, and sulphamethoxazole) based on the specific inhibitors for different enzyme subtypes. The final volume of the incubation system was 200 μL (Shi et al., 2020), consisting of RLM (0.5 mg/mL), NADPH, PBS buffer solution, and a cocktail probe drug mixture. Preincubation was conducted for 5 min in a water bath at 37°C, after which NADPH (1 mmol/L) was added, and incubation continued for 30 min at 37°C. Once the incubation period elapsed, the reaction was terminated by the addition of an ice-cold IS-containing acetonitrile solution. The samples were vortexed for 2 min and centrifuged at 15,000 RPM for 10 min. The resulting supernatant was filtered through a 0.22-μm organic microporous membrane, and 20 μL of the filtered solution was injected into the HPLC assay. To prevent the organic solvents from inducing or inhibiting the enzyme, the volume fraction of organic solvents in the entire incubation system was maintained at 1%. In the inactive control group, NADPH was omitted to determine the maximum substrate concentration. The inhibitor group was added with corresponding inhibitor, while all other procedures remained identical.

### 2.8 Study on the detoxification mechanism of Zhicaowu–Hezi pairing

#### 2.8.1 Biochemical and histopathological analyses

After the final administration, all rats were euthanized, and blood and liver samples were collected. The levels of biochemical indicators ALT, AST and ALP in the blood were detected according to the kit instruction. The liver histopathology was analyzed by H&E staining. Briefly, liver tissues were fixed in 10% paraformaldehyde (for over 24 h, 4°C), paraffin-embedded, and sectioned into 5-µm slices). Following deparaffinization with xylene and ethanol dehydration (90%, 80%, 70%), sections were H&E-stained and imaged under a biomicroscope (Sunny Instruments Co., Ltd., Ningbo, China).

#### 2.8.2 RT-qPCR analysis

Total RNA was extracted from the rat liver tissue using TRNzol Universal Reagent. The concentration and quality of the extracted RNA were determined at 260 nm and 280 nm using an ultra-micro UV spectrophotometer (METTLER TOLEDO Co., Ltd., Shanghai, China). RNA from each group was diluted according to the FastKing gDNA Dispelling RT SuperMix kit instruction, and subsequent reverse transcription was performed in accordance with the manufacturer’s protocols. The total volume of the reaction was 20 μL, and the specific procedure was as follows: 2 μL of 5× FastKing-RT SuperMix and 4 μL of total RNA, replenished to 20 μL with RNase-free H_2_O, were centrifuged and thorough mixing. The solution was then incubated at 42°C for 15 min and 95°C for 3 min in a gradient PCR apparatus. Gene primers were synthesized by Sangyo Bioengineering Co. The specific primer sequences are provided in Supplementary Table S1 and were sourced from Shanghai-Based Company, Ltd. RT-qPCR was conducted using a real-time fluorescent quantitative PCR instrument (Applied Biosystems) in accordance with the instructions provided with the SYBR Green PCR kit. The total reaction system included: 20 μL 2× FastReal qPCR PreMix, 10 μL of cDNA, 0.6 μL of forward primer (10 μM), 0.6 μL of reverse primer (10 μM), 2 μL of 50× ROX Reference Dye, and 2 μL of RNase-free H_2_O, resulting in a total volume of 20 μL. The reaction conditions were as follows: pre-denaturation at 95°C for 2 min, followed by 40 cycles at 60°C for 15 s. The melting curve was constructed at 95°C for 15 min and then at 95°C for 15 s. A lysis curve was constructed at 95°C for 15 s and 60°C for 1 min. The mRNA relative expression level was evaluated using the 2^−ΔΔCT^ value.

### 2.9 Statistical analysis

The experimental data were statistically analyzed using IBM SPSS Statistics 26 and GraphPad Prism 8.3.0. Groups comparisons were made via *t*-tests, and the results of the experiments were expressed as mean ± standard deviation (SD). Statistical significance was defined as *P* < 0.05.

## 3 Results

### 3.1 Impact of zhicaowu-hezi pairing on CYP450 enzyme activity in rats *in vivo*

#### 3.1.1 Specificity

**FIGURE 1:**
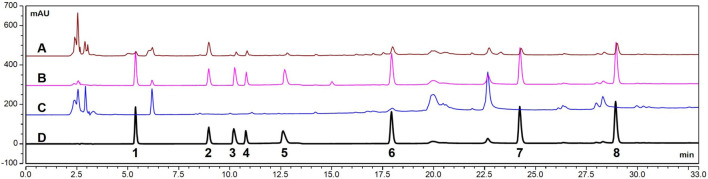
HPLC chromatograms of seven probe drugs in plasma. **(A)**. Plasma samples (with IS) obtained from rats after oral administration of mixed probe drugs. **(B)**. Blank plasma spiked with a mixture of probe drug and IS. **(C)**. Blank plasma. **(D)**. Mixed probe drugs (with IS). (1. theophylline, 2. IS, 3. metoprolol, 4. omeprazole, 5. bupropion, 6. chlorzoxazone, 7. testosterone, 8. diclofenac).

The HPLC chromatograms of the seven probe drugs in plasma are presented in Figure 1. Each probe drug was effectively separated, and neither endogenous substances nor the IS interfered with the sample analysis.

#### 3.1.2 Standard curve

**FIGURE 2:**
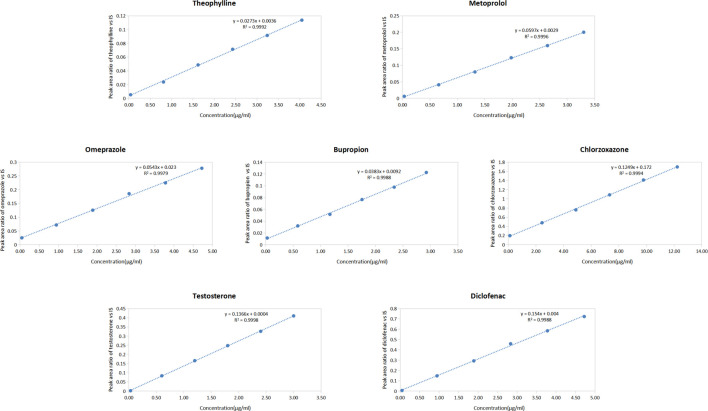
Standard curves of seven probe drugs in rat plasma (Including the regression equation and the correlation coefficient) (n = 3) (y = peak area ratio of probe drugs vs. IS; x = concentration of probe drugs).

Linear equations were established based on the peak area ratio of the probe drug to that of the IS and the concentration of each probe drug in rat plasma. The linear equations for the seven probe drugs in the plasma are presented in Figure 2. The results demonstrated excellent linearity within the concentration range, with correlation coefficients (*R* ^2^) exceeding 0.997 for each probe drug.

#### 3.1.3 Precision

The peak area ratio of each probe drug to that of the IS was substituted into the linear equation, and the intra- and inter-day precision of the seven probe drugs was calculated using the measured concentrations (Table 1). The findings demonstrated that the intra- and inter-day precision of each probe drug was within 15%, meeting the acceptance criteria for biological sample analysis.

**TABLE 1:** Precision of seven probe drugs in rat plasma (‾x±SD, n = 5).

Probe drug	Concentration (μg/mL)	Intra-day precision		Inter-day precision	
		‾Measured concentration (μg/mL)	RSD (%)	‾Measured concentration (μg/mL)	RSD (%)
theophylline	0.81	0.73 ± 0.01	1.84	0.82 ± 0.10	12.68
	2.43	2.52 ± 0.11	4.45	2.57 ± 0.13	5.19
	4.05	4.36 ± 0.35	7.96	4.63 ± 0.59	12.82
metoprolol	0.66	0.64 ± 0.01	1.49	0.67 ± 0.06	9.21
	1.98	1.95 ± 0.08	4.24	1.90 ± 0.09	4.65
	3.30	3.16 ± 0.14	4.41	3.15 ± 0.13	4.18
omeprazole	0.95	0.85 ± 0.04	4.20	0.86 ± 0.08	9.64
	2.84	2.68 ± 0.24	8.91	2.85 ± 0.25	8.94
	4.73	4.60 ± 0.12	2.69	4.75 ± 0.11	2.29
bupropion	0.59	0.56 ± 0.03	5.78	0.53 ± 0.05	10.12
	1.76	1.82 ± 0.02	1.21	1.86 ± 0.07	3.64
	2.93	2.61 ± 0.02	5.18	2.94 ± 0.17	5.66
chlorzoxazone	2.45	2.47 ± 0.21	8.44	2.70 ± 0.20	7.23
	7.34	7.67 ± 0.56	7.30	7.00 ± 0.30	4.24
	12.23	11.82 ± 0.37	3.12	12.30 ± 0.49	3.96
testosterone	0.60	0.56 ± 0.07	12.04	0.56 ± 0.05	8.38
	1.80	1.77 ± 0.07	4.16	1.85 ± 0.18	9.46
	3.00	2.99 ± 0.07	2.39	2.76 ± 0.24	8.60
diclofenac	0.95	0.94 ± 0.05	5.73	0.96 ± 0.06	6.63
	2.84	3.10 ± 0.21	6.81	3.10 ± 0.24	7.91
	4.73	4.54 ± 0.12	2.64	4.89 ± 0.26	5.36

#### 3.1.4 Extraction recovery

The peak area ratio of each probe drug to the IS was applied to the standard curve, and the relative and absolute recoveries of the seven probe drugs were calculated using the measured concentrations. Table 2 shows the relative and absolute recoveries of the seven probe drugs in plasma. The relative and absolute recoveries of the probe drugs at three concentrations levels were all >75%, meeting the requirements for the analysis of biological samples.

$$\text{Relative Recovery }\left(\%\right)=\left(\text{Measured concentration }/\text{ Nominal spiked concentration}\right)\times 100\%$$

$$\text{Absolute Recovery }\left(\%\right)=\left[\left(\text{Peak }{\text{Area}}_{\text{Analyte},\text{spiked}\;\text{sample}}\;/\text{ Peak }{\text{Area}}_{\text{IS},\text{spiked}\;\text{sample}}\right)\;/\;\left(\text{Peak }{\text{Area}}_{\text{Analyte},\text{neat}\;\text{standard}}\;/\text{ Peak }{\text{Area}}_{\text{IS},\text{neat}\;\text{standard}}\right)\right]\times 100\%$$

**TABLE 2:** Extraction recovery of seven probe drugs in rat plasma (‾x±SD, n = 5).

Probe drug	Nominal concentration (μg/mL)	Relative recovery		Absolute recovery	
		‾X ± SD (%)	RSD (%)	‾X ± SD (%)	RSD (%)
theophylline	0.81	90.78 ± 1.18	1.30	86.23 ± 5.24	6.08
	2.43	100.78 ± 1.30	1.29	89.37 ± 1.78	1.99
	4.05	101.75 ± 6.48	6.37	81.99 ± 2.42	2.95
metoprolol	0.66	97.03 ± 0.14	0.15	94.65 ± 11.81	12.47
	1.98	99.41 ± 4.30	4.32	99.71 ± 0.38	0.38
	3.30	98.68 ± 3.00	3.04	97.86 ± 2.78	2.84
omeprazole	0.95	91.31 ± 3.90	4.27	84.91 ± 9.14	10.76
	2.84	92.40 ± 7.44	8.05	86.26 ± 3.98	4.61
	4.73	98.67 ± 0.57	0.58	95.70 ± 2.30	2.40
bupropion	0.59	95.08 ± 5.03	5.29	97.81 ± 2.83	2.89
	1.76	102.20 ± 3.25	3.18	91.70 ± 6.11	6.67
	2.93	92.08 ± 3.68	3.99	91.84 ± 3.20	3.49
chlorzoxazone	2.45	101.24 ± 6.21	6.14	89.58 ± 4.32	4.82
	7.34	97.81 ± 4.04	4.13	89.55 ± 1.33	1.49
	12.23	98.56 ± 2.53	2.56	98.84 ± 4.09	4.14
testosterone	0.60	91.07 ± 12.86	14.12	94.41 ± 2.16	2.28
	1.80	103.27 ± 6.44	6.23	96.57 ± 4.26	4.41
	3.00	100.50 ± 2.58	2.57	80.20 ± 3.75	4.68
diclofenac	0.95	99.93 ± 5.22	5.23	97.03 ± 1.03	1.07
	2.84	107.37 ± 6.66	6.21	79.76 ± 5.78	7.24
	4.73	97.72 ± 2.16	2.21	91.86 ± 6.81	7.42

#### 3.1.5 Pharmacokinetic study

**FIGURE 3:**
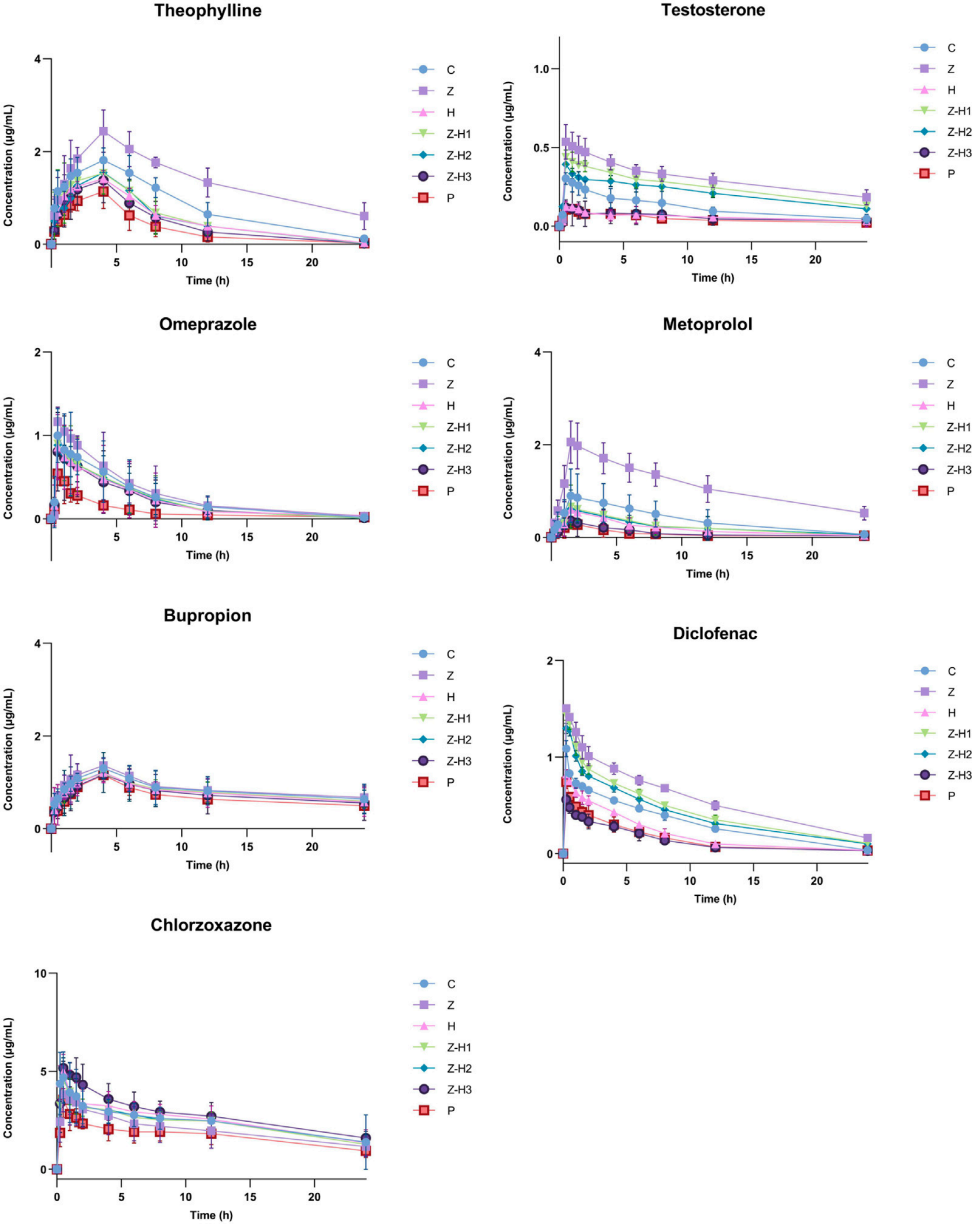
Blood concentration-time curves for theophylline, metoprolol, omeprazole, bupropion, chlorzoxazone, testosterone and diclofenac.

First, an *in vivo* evaluation was performed to assess the effect of the Zhicaowu–Hezi pairing on the primary pharmacokinetic parameters of the seven cocktail probe drugs in rat plasma, namely, area under the curve (AUC_(0-t)_, AUC_(0-∞)_)), terminal half-life (t_1/2_ z), plasma clearance (CLz/F), and peak concentration (C_max_) (Table 3). Figure 3 illustrates the blood concentration-time curves of theophylline, metoprolol, omeprazole, bupropion, chlorzoxazone, testosterone, and diclofenac in rats after intragastric administration of a mixed solution of the seven cocktail probe drugs.

**TABLE 3:** Pharmacokinetic parameters of seven probe drugs (‾x±SD, n = 8).

Probe drug	Group	AUC_(0-t)_ (μg/ml*h)	AUC_(0-∞)_ (μg/ml*h)	t_1/2_ z (h)	CLz/F (mL/h/kg)	C_max_ (μg/mL)
theophylline	C	21.860 ± 1.253	22.543 ± 1.437	5.273 ± 0.526	465.260 ± 71.379	1.811 ± 0.094
	Z	27.137 ± 1.895***	28.662 ± 1.591***	9.995 ± 1.050***	293.035 ± 37.399***	2.437 ± 0.057***
	H	18.537 ± 0.937**	19.381 ± 1.281**	3.568 ± 0.190**	592.648 ± 31.273**	1.410 ± 0.185**
	Z-H1	19.199 ± 0.653**	19.736 ± 1.054**	3.874 ± 0.642**	375.912 ± 20.382**	1.542 ± 0.144*
	Z-H2	18.818 ± 0.620**	19.534 ± 0.005*	3.851 ± 0.312**	584.239 ± 18.163**	1.557 ± 0.131*
	Z-H3	17.061 ± 1.445***	18.001 ± 1.538***	3.709 ± 0.186**	616.387 ± 23.645***	1.384 ± 0.115***
	P	12.257 ± 0.742***	13.565 ± 0.801***	2.509 ± 0.436***	7,744.326 ± 56.253***	1.138 ± 0.201***
metoprolol	C	9.603 ± 0.988	10.097 ± 1.484	7.951 ± 1.152	1,580.058 ± 223.962	0.891 ± 0.114
	Z	15.781 ± 1.584***	16.972 ± 1.130***	11.888 ± 1.294***	753.952 ± 97.661***	2.053 ± 0.132***
	H	5.891 ± 0.529***	6.003 ± 0.635***	4.017 ± 0.255***	1804.051 ± 167.180*	0.597 ± 0.078***
	Z-H1	6.001 ± 0.814***	6.182 ± 0.731***	5.591 ± 0.718***	1788.311 ± 137.404*	0.661 ± 0.062***
	Z-H2	5.918 ± 0.609***	6.103 ± 0.570***	4.937 ± 0.587***	1797.830 ± 66.013*	0.610 ± 0.087***
	Z-H3	3.650 ± 0.294***	4.071 ± 0.348***	2.817 ± 0.187***	2,471.151 ± 225.361***	0.356 ± 0.048***
	P	3.511 ± 0.445***	3.623 ± 0.461***	2.532 ± 0.221***	2,495.074 ± 157.841***	0.312 ± 0.041***
omeprazole	C	3.843 ± 0.372	3.896 ± 0.394	4.689 ± 0.467	2,606.225 ± 208.442	0.995 ± 0.078
	Z	4.191 ± 0.351	4.265 ± 0.322	4.948 ± 0.422	2,520.415 ± 278.681	1.164 ± 0.127*
	H	3.506 ± 0.485	3.567 ± 0.477	4.037 ± 0.400*	2,789.835 ± 150.866	0.858 ± 0.130*
	Z-H1	3.772 ± 0.482	3.929 ± 0.505	4.701 ± 0.421	2,757.704 ± 387.945	0.899 ± 0.125
	Z-H2	3.750 ± 0.153	3.827 ± 0.420	4.590 ± 0.404	2,799.647 ± 310.234	0.877 ± 0.048
	Z-H3	3.570 ± 0.280	3.643 ± 0.358	3.942 ± 0.395*	2,834.456 ± 273.996	0.791 ± 0.101**
	P	3.040 ± 0.177**	3.187 ± 0.239**	2.761 ± 0.227***	3,028.688 ± 253.193*	0.535 ± 0.063***
bupropion	C	15.819 ± 1.316	16.340 ± 1.407	18.743 ± 0.948	429.580 ± 59.098	1.299 ± 0.093
	Z	16.357 ± 1.872	17.046 ± 1.566	19.590 ± 0.952	407.926 ± 55.463	1.359 ± 0.126
	H	14.356 ± 0.370	15.157 ± 0.594	17.684 ± 0.604	479.806 ± 43.311	1.209 ± 0.062
	Z-H1	14.818 ± 2.207	15.831 ± 1.701	17.884 ± 0.824	436.396 ± 35.719	1.181 ± 0.179
	Z-H2	14.581 ± 1.011	14.937 ± 1.069	17.650 ± 0.531	466.372 ± 26.817	1.218 ± 0.159
	Z-H3	14.200 ± 1.106	14.697 ± 0.814*	17.096 ± 2.409*	481.696 ± 33.422	1.173 ± 0.160
	P	13.249 ± 1.396**	13.714 ± 1.057**	15.564 ± 0.555***	452.051 ± 75.378	1.151 ± 0.138
chlorzoxazone	C	40.746 ± 2.056	53.614 ± 3.550	16.969 ± 2.150	560.148 ± 89.556	4.689 ± 0.325
	Z	36.357 ± 2.942	47.158 ± 9.757	14.042 ± 2.720	709.955 ± 37.134**	3.823 ± 0.347*
	H	43.073 ± 3.271	58.267 ± 4.837	18.234 ± 3.615	461.709 ± 48.863*	4.976 ± 0.362
	Z-H1	39.451 ± 6.113	51.352 ± 5.148	15.119 ± 2.473	678.317 ± 77.552*	4.499 ± 0.493
	Z-H2	40.408 ± 5.254	55.643 ± 5.494	15.866 ± 2.706	629.281 ± 100.941	4.641 ± 0.502
	Z-H3	46.506 ± 8.342	66.765 ± 7.960**	19.649 ± 3.124	345.876 ± 25.800***	5.168 ± 0.463
	P	32.754 ± 4.037**	47.632 ± 6.051	13.368 ± 1.308*	804.366 ± 66.616***	3.543 ± 0.431***
testosterone	C	1.210 ± 0.081	1.407 ± 0.069	11.127 ± 1.110	4,007.587 ± 576.688	0.295 ± 0.054
	Z	1.838 ± 0.112***	2.040 ± 0.205***	17.043 ± 0.939***	2,796.981 ± 321.286***	0.542 ± 0.099***
	H	0.847 ± 0.050***	0.949 ± 0.097***	8.988 ± 0.768*	5,005.686 ± 647.378**	0.129 ± 0.018***
	Z-H1	1.699 ± 0.092***	1.840 ± 0.098***	15.227 ± 1.537***	3,157.447 ± 353.533*	0.440 ± 0.041**
	Z-H2	1.571 ± 0.087***	1.666 ± 0.179**	14.318 ± 2.081**	3,782.662 ± 560.228	0.386 ± 0.076*
	Z-H3	0.795 ± 0.048***	0.916 ± 0.106***	8.115 ± 1.083**	5,271.776 ± 520.452***	0.117 ± 0.012***
	P	0.756 ± 0.048***	0.907 ± 0.093***	7.394 ± 0.958***	6,479.436 ± 351.908***	0.107 ± 0.015***
diclofenac	C	5.233 ± 0.465	5.474 ± 0.358	4.982 ± 0.557	2,535.135 ± 294.979	1.084 ± 0.115
	Z	6.790 ± 0.985***	6.882 ± 1.119**	6.978 ± 0.701***	2042.408 ± 206.113*	1.508 ± 0.095***
	H	4.361 ± 0.524*	4.522 ± 0.459*	4.018 ± 0.773*	2,944.398 ± 363.305	0.817 ± 0.105***
	Z-H1	6.439 ± 0.924**	6.723 ± 0.750**	5.800 ± 0.761*	2,100.150 ± 292.459	1.434 ± 0.105***
	Z-H2	6.132 ± 0.426*	6.389 ± 0.481*	5.772 ± 0.636*	2,128.872 ± 304.503	1.301 ± 0.101***
	Z-H3	3.879 ± 0.476***	3.966 ± 0.615***	3.787 ± 0.492**	3,379.090 ± 313.173***	0.557 ± 0.041***
	P	3.978 ± 0.385**	4.057 ± 0.343***	3.342 ± 0.425***	3,071.310 ± 519.261*	0.735 ± 0.068***

Comparing medication groups with the group C, *P < 0.05, **P < 0.01,***P < 0.001.

For CYP1a2 (theophylline) and CYP2d2 (metoprolol), Zhicaowu alone showed strong inhibitory effects, evidenced by significantly increased AUC_(0-t)_, AUC_(0-∞)_), and C_max_ (all *P* < 0.001), prolonged t_1/2_ z (*P* < 0.001), and decreased CLz/F (*P* < 0.001). However, when combined with Hezi, these effects were reversed, indicating clear induction of both enzymes. The degree of induction varied with the mixing ratio, with Z-H3 (1:3) showing the most significant effects.

Interestingly, the combination exhibited differential effects on CYP3a1 (testosterone) and CYP2c11 (diclofenac) depending on the ratio. While Zhicaowu alone inhibited both enzymes, the Z-H1 and Z-H2 combinations maintained this inhibition, whereas the Z-H3 combination shifted to induction. This ratio-dependent modulation suggests complex herb-herb interactions.

For CYP2e1 (chlorzoxazone), only minor effects were observed except in the Z-H3 group, which showed significant inhibition. No changes were detected for CYP2b1 (bupropion) or CYP2c13 (omeprazole) substrates, indicating these isoforms were unaffected by the treatments.

### 3.2 Effect of Zhicaowu–Hezi pairing on CYP450 enzyme activity in RLM

#### 3.2.1 Specificity

**FIGURE 4:**
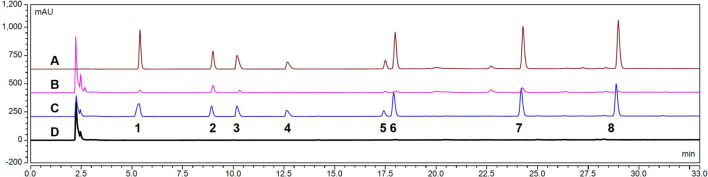
High-performance liquid chromatography (HPLC) chromatogram of seven probe drugs in rat liver microsomes (RLM): **(A)** Mixed probe drug standard (including internal standard [IS]) **(B)**. RLM samples (including IS) after incubation **(C)**. Blank RLM mixed with the probe drug and IS **(D)**. Blank RLM. (1. Theophylline; 2. IS; 3. metoprolol; 4. bupropion; 5. mephenytoin; 6. chlorzoxazone; 7. testosterone; 8. diclofenac).

The HPLC chromatogram of probe drugs is shown in Figure 4. The probe drugs—inlcuding theophylline, metoprolol, bupropion, mephenytoin, chlorzoxazone, testosterone, and diclofenac—showed a good separation effect. Additionally, neither the compounds in RLM nor the IS interfered with the sample analysis results.

#### 3.2.2 Standard curve

**FIGURE 5:**
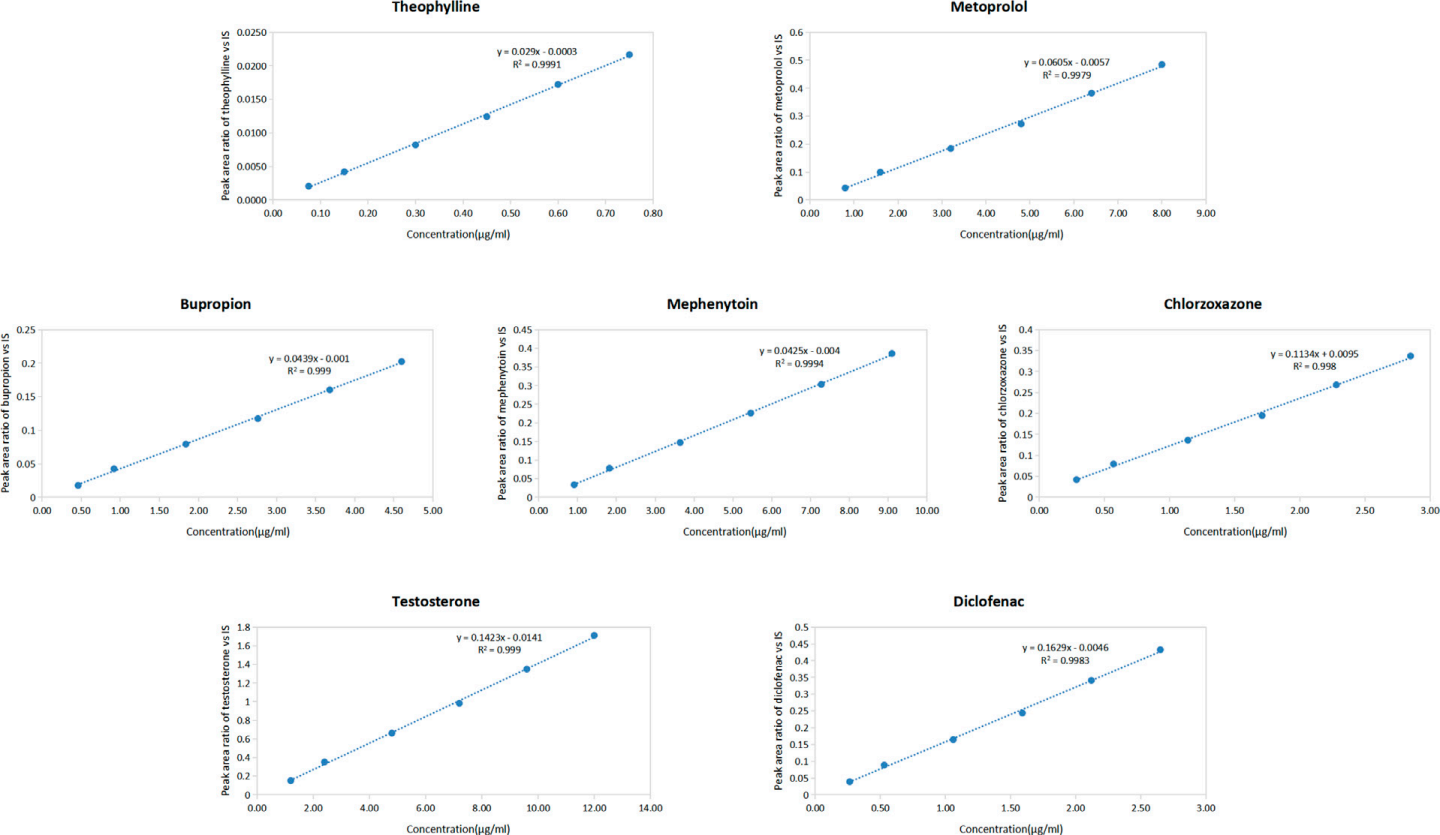
Standard curves of probe drugs in rat liver microsomes (RLM) (n = 3).

The standard curve equations of probe drugs are shown in Figure 5. The results indicate a strong linear relationship between probe drugs, with a correlation coefficient *R* ^2^ > 0.997.

#### 3.2.3 Precision

The intra-day and inter-day precision for the probe drug at low, medium, and high concentrations are shown in Table 4. The results showed that the intra-day and inter-day precision of each probe drug was below 15%, meeting the requirements of biological sample detection.

**TABLE 4:** Precision of seven probe drugs in rat liver microsomes (RLM) (x±SD, n = 5).

Probe drug	Nominal concentration (μg/mL)	Intra-day precision		Inter-day precision	
		Measured concentration (μg/mL)	RSD (%)	Measured concentration (μg/mL)	RSD (%)
Theophylline	0.15	0.16 ± 0.01	4.35	0.17 ± 0.02	9.98
	0.45	0.43 ± 0.02	4.71	0.48 ± 0.06	12.76
	0.75	0.75 ± 0.01	1.32	0.78 ± 0.09	11.81
Metoprolol	1.60	1.71 ± 0.03	1.83	1.64 ± 0.06	3.83
	4.80	4.56 ± 0.13	2.77	4.49 ± 0.13	2.82
	8.00	7.99 ± 0.10	1.28	7.73 ± 0.37	4.77
Bupropion	0.92	0.98 ± 0.01	1.34	0.95 ± 0.04	4.16
	2.76	2.66 ± 0.04	1.57	2.62 ± 0.09	3.28
	4.60	4.65 ± 0.04	0.78	4.43 ± 0.24	5.34
Mephenytoin	1.82	1.91 ± 0.03	1.47	1.86 ± 0.05	2.79
	5.46	5.35 ± 0.19	3.53	5.24 ± 0.16	3.05
	9.10	9.16 ± 0.09	0.97	8.86 ± 0.40	4.57
Chlorzoxazone	0.57	0.60 ± 0.02	2.66	0.59 ± 0.02	3.43
	1.71	1.66 ± 0.06	3.71	1.67 ± 0.05	2.91
	2.85	2.86 ± 0.03	1.02	2.78 ± 0.11	4.10
Testosterone	2.40	2.54 ± 0.04	1.54	2.45 ± 0.08	3.25
	7.20	6.99 ± 0.09	1.26	6.85 ± 0.21	3.02
	12.00	12.17 ± 0.10	0.80	11.80 ± 0.59	5.02
Diclofenac	0.53	0.56 ± 0.01	2.54	0.57 ± 0.03	5.17
	1.59	1.54 ± 0.03	1.71	1.54 ± 0.04	2.72
	2.65	2.67 ± 0.02	0.91	2.58 ± 0.11	4.17

#### 3.2.4 Extraction recovery

The relative and absolute recoveries of low, medium, and high-concentration probe drugs in RLM are shown in Table 5. The relative and absolute recoveries of the three concentrations of probe drugs exceeded 75%, meeting the acceptance criteria of biological sample analysis.

**TABLE 5:** Extraction recovery of seven probe drugs in rat liver microsomes (RLM) (x±SD, n = 5).

Probe drug	Nominal concentration (μg/mL)	Relative recovery		Absolute recovery	
		‾X ± SD (%)	RSD (%)	‾X ± SD (%)	RSD (%)
	0.15	107.11 ± 5.95	5.55	88.24 ± 4.23	4.80
	0.45	95.79 ± 1.80	1.87	97.57 ± 1.48	1.52
Theophylline	0.75	100.94 ± 0.82	0.81	94.61 ± 6.05	6.39
	1.60	106.63 ± 3.51	3.29	95.09 ± 1.63	1.72
	4.80	95.80 ± 0.53	0.55	96.17 ± 2.08	2.16
Metoprolol	8.00	100.34 ± 0.90	0.89	89.88 ± 2.56	2.85
	0.92	107.59 ± 1.22	1.13	93.24 ± 4.48	5.20
	2.76	96.61 ± 1.04	1.08	96.09 ± 4.09	4.26
Bupropion	4.60	101.24 ± 0.39	0.38	76.64 ± 0.92	1.20
	1.82	105.46 ± 1.27	1.20	89.94 ± 2.04	2.26
	5.46	97.23 ± 1.85	1.91	87.60 ± 3.87	4.42
Mephenytoin	9.10	101.05 ± 0.31	0.31	88.12 ± 0.85	0.97
	0.57	106.26 ± 3.61	3.40	93.17 ± 6.34	6.80
	1.71	95.65 ± 0.29	0.30	92.14 ± 6.65	7.21
Chlorzoxazone	2.85	100.41 ± 0.55	0.55	88.22 ± 2.31	2.62
	2.40	106.31 ± 1.32	1.24	92.02 ± 8.69	9.44
	7.20	96.68 ± 0.94	0.97	96.75 ± 1.49	1.54
Testosterone	12.00	101.62 ± 0.53	0.53	90.72 ± 0.49	0.43
	0.53	104.92 ± 3.59	3.42	80.11 ± 2.97	3.71
	1.59	96.00 ± 0.28	0.29	92.39 ± 5.05	5.47
Diclofena	2.65	101.04 ± 0.20	0.20	86.33 ± 1.42	1.64

#### 3.2.5 Investigation of protein concentration in RLM

The protein concentration of the prepared RLM was determined using a BCA protein assay kit. The standard curve exhibited a linear regression equation of y = 0.6553x+0.44, with a correlation coefficient (*R* ^2^) of 0.9985. The measured protein concentrations of RLM across all groups ranged from 3.33 to 3.53 mg/mL. No significant inter-group differences were observed (*p* > 0.05), ensuring consistent enzyme loading in subsequent activity assays.

#### 3.2.6 Effects of specific inhibitors on corresponding CYP450 enzymes

The effects of specific inhibitors on CYP450 enzyme activities in RLM are shown in Table 6. Compared with that in the blank control group, the metabolic clearance rate of the corresponding probe drugs for CYP450 enzymes was significantly decreased in each inhibitor group (***P < 0.001), indicating CYP450 enzyme inhibition and confirming the reliability of the experiment.

**TABLE 6:** Effects of inhibitors on CYP450 enzyme activity in rat liver microsomes (RLM) (x±SD, n = 3).

Group	CYP1a2 (%)	CYP2d2 (%)	CYP2b1(%)	CYP2c13(%)	CYP2e1 (%)	CYP3a1 (%)	CYP2c11(%)
Blank control	36.29 ± 1.14	12.58 ± 2.12	7.92 ± 0.60	15.97 ± 0.68	15.80 ± 0.34	25.04 ± 1.93	21.12 ± 3.80
α-naphthoflavone	28.92 ± 1.91***	13.12 ± 0.82	8.00 ± 0.97	16.65 ± 3.04	15.13 ± 1.76	25.69 ± 2.09	23.02 ± 0.55
Quinidine	37.43 ± 1.01	5.56 ± 0.52***	8.10 ± 1.52	15.61 ± 0.69	16.06 ± 1.23	25.93 ± 0.05	22.67 ± 2.68
Thiotepa	35.91 ± 1.40	12.67 ± 0.65	2.24 ± 0.16***	14.43 ± 0.36	15.20 ± 0.20	25.72 ± 2.36	21.84 ± 2.64
Ticlopidine	36.19 ± 1.75	12.25 ± 0.69	8.64 ± 0.80	4.02 ± 0.61***	15.67 ± 0.59	25.24 ± 2.17	21.74 ± 0.76
4-methylpyrazole	36.27 ± 1.05	12.64 ± 0.83	7.18 ± 0.80	16.82 ± 0.52	6.32 ± 0.58***	25.57 ± 2.18	22.44 ± 0.64
Ketoconazole	36.04 ± 1.71	12.73 ± 1.11	7.11 ± 0.17	17.26 ± 1.08	15.40 ± 0.67	5.45 ± 2.49***	22.50 ± 0.83
Sulfaphenazolum	37.16 ± 0.71	13.50 ± 0.30	7.74 ± 0.98	16.47 ± 1.34	16.11 ± 0.18	25.69 ± 1.69	6.16 ± 0.65***

Compared with blank control group, ***P < 0.001.

#### 3.2.7 Metabolic clearance study

**FIGURE 6:**
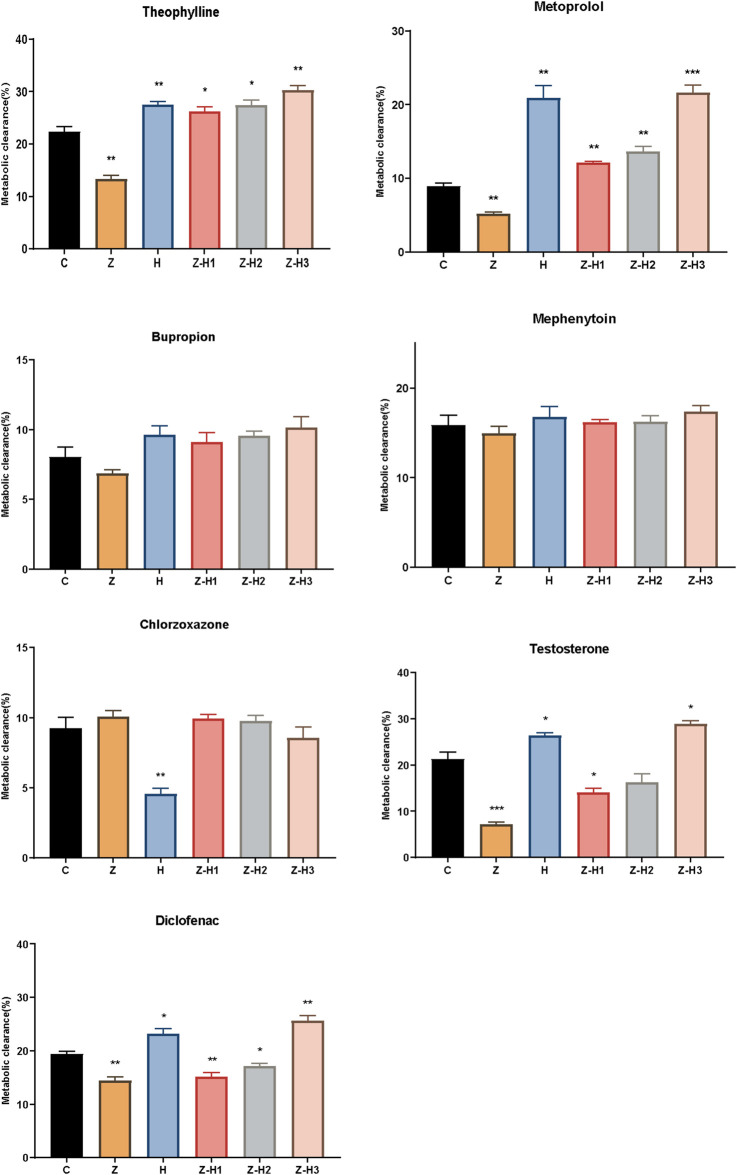
Metabolic clearance of seven probe drugs (theophylline, metoprolol, bupropion, mephenytoin, chlorzoxazone, testosterone, and diclofenac) in different groups. Data are expressed as means ± SD (n = 3). Comparing medication groups with the group C, *P < 0.05, **P < 0.01,***P < 0.001.

The effect of Zhicaowu–Hezi on CYP450 activity in RLM was evaluated *in vitro*. Compared to Group C, Group Z showed significantly decreased metabolic clearances of theophylline, metoprolol, testosterone, and diclofenac (**P < 0.01 or ***P < 0.001) (Figure 6). In contrast, the metabolic clearances of theophylline and metoprolol were significantly increased in Groups H, Z-H1, Z-H2, and Z-H3 (*P < 0.05, **P < 0.01 or ***P < 0.001). For testosterone and diclofenac, metabolic clearances were higher in Groups H and Z-H3 (*P < 0.05 or **P < 0.01) but lower in Groups Z-H1 and Z-H2 (*P < 0.05 or **P < 0.01). No significant changes were observed in the metabolic clearances of bupropion, mephenytoin, and chlorzoxazone across all Zhicaowu-Hezi paired groups (*P* > 0.05).

These results indicate that Zhicaowu alone inhibited the activities of CYP1a2, CYP2d2, CYP3a1, and CYP2c11, whereas its combination with Hezi induced these enzymes to varying degrees. However, the Zhicaowu-Hezi combination had no effect on CYP2b1, CYP2c13, and CYP2e1 activities. These findings are consistent with the *in vivo* results.

### 3.3 Effect of the Zhicaowu–Hezi combination on rat liver histopathology

**FIGURE 7:**
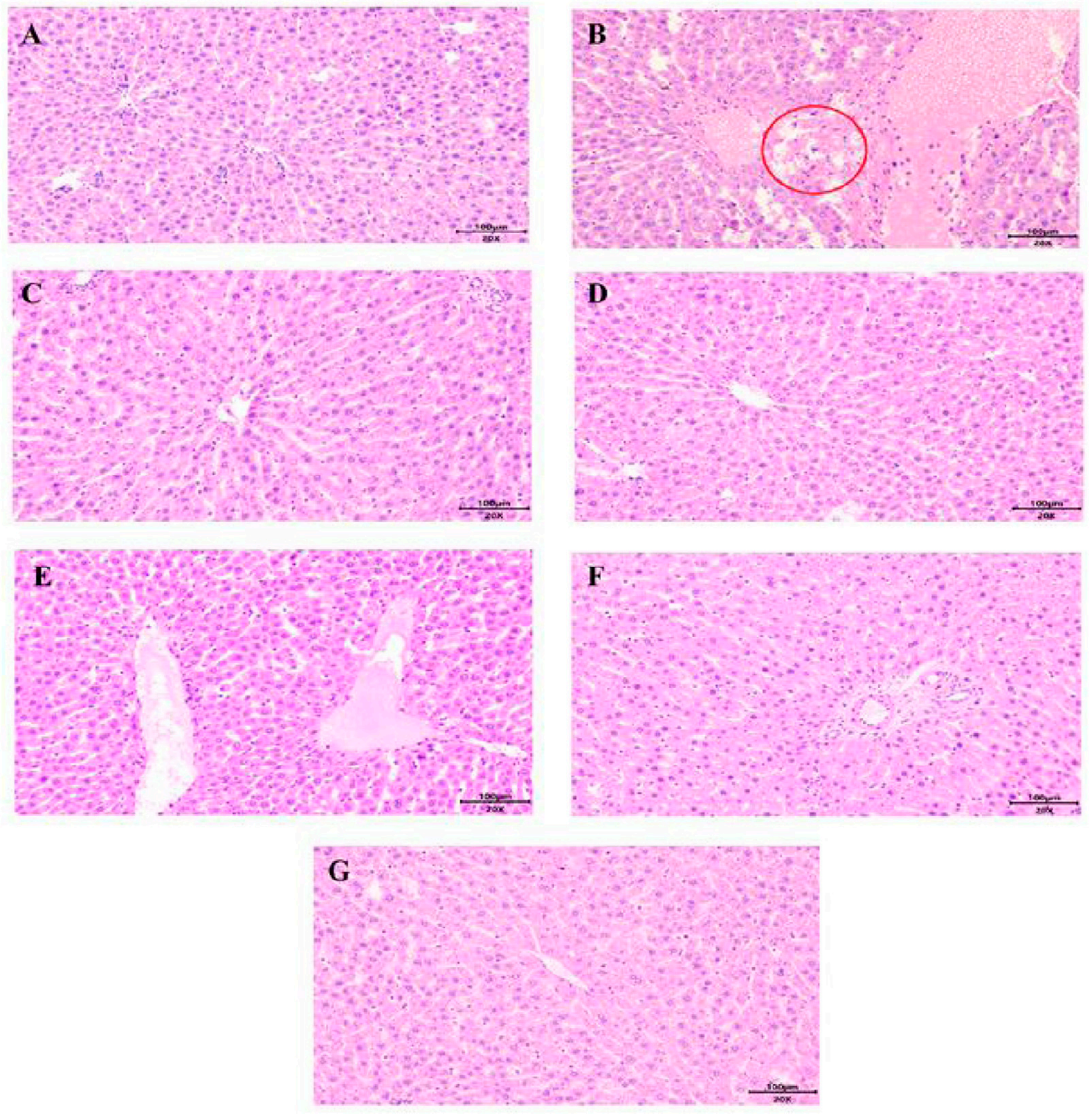
Histopathological examination of rat livers from different groups (Bar = 100 µm) **(A)** C, **(B)** Z, **(C)** H, **(D)** Z-H1, **(E)** Z-H2, **(F)** Z-H3, **(G)** P. Focal punctate necrosis (red circle).

The histopathological examination of liver tissue showed that hepatocytes around the central vein (lobular zone) in group Z presented focal punctate necrosis, accompanied by mild lymphocyte infiltration and scattered eosinophilic hepatocytes (Figure 7), suggesting early-stage toxic injury. In contrast, the hepatocytes of rats in groups C, H, Z-H1, Z-H2 and Z-H3 were neatly arranged with clear structure integrity. No signs of necrosis, degeneration or inflammatory cell infiltration was observed in the liver tissue cells. Scattered cellular edema could be observed in group P. Hence, Hezi may mitigate the hepatotoxicity of Zhicaowu.

### 3.4 Effect of the Zhicaowu–Hezi combination on the biochemical indices of rat

**FIGURE 8:**
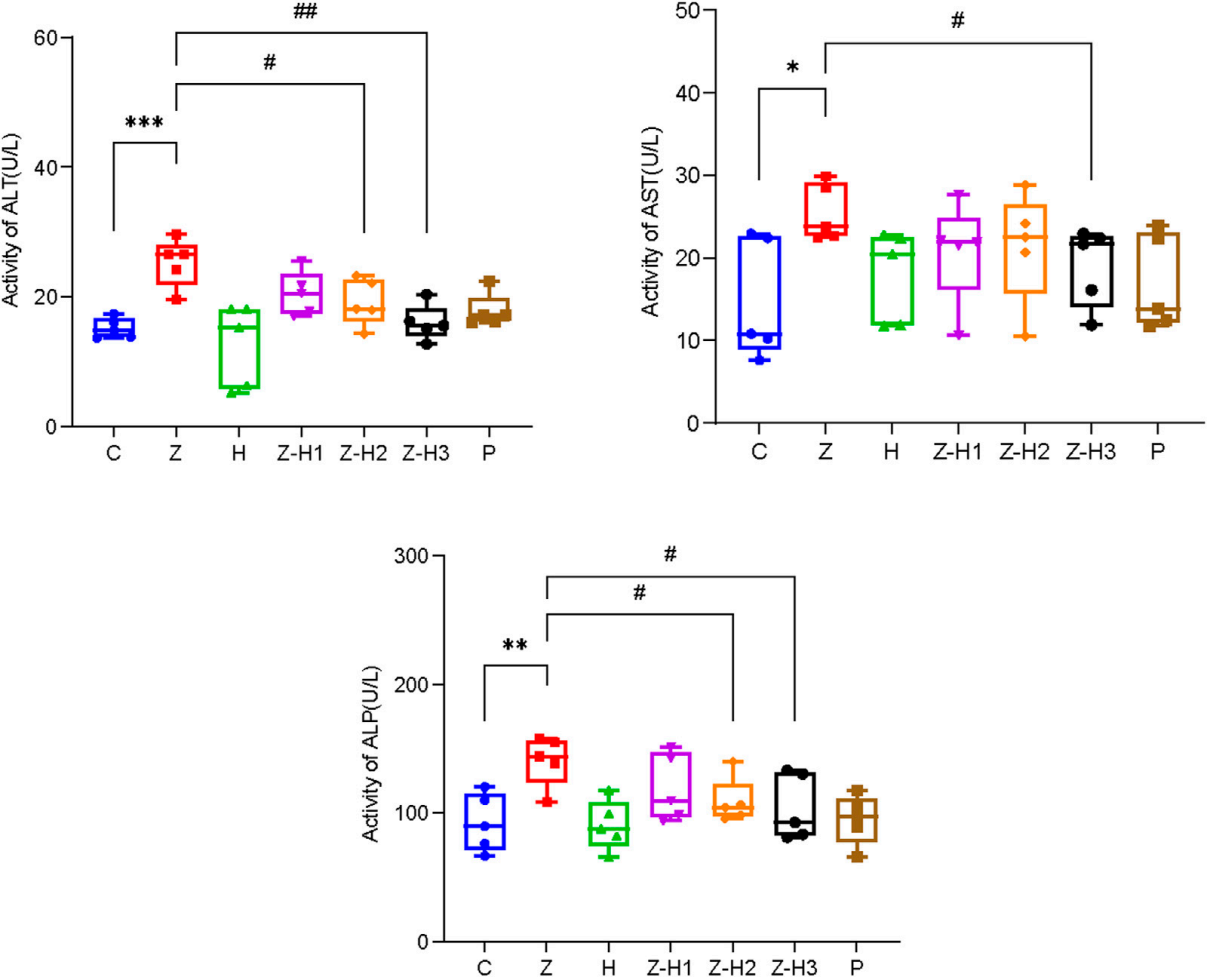
Changes in the plasma levels of alanine aminotransferase (ALT), aspartate aminotransferase (AST), and alanine phosphatase (ALP) in different rat groups. Data are expressed as means ± SD. (n = 5). Comparing medication groups with the group C, *P < 0.05, **P < 0.01,***P < 0.001. Comparing medication groups with the group Z, #*P* < 0.05, ##*P* < 0.01.

Compared with Group C, plasma levels of ALT, AST, and ALP were significantly elevated in Group Z (*P < 0.05, **P < 0.01, or***P < 0.001) (Figure 8). In contrast, plasma ALT, AST, and ALP levels were significantly lower in the Z-H2 and Z-H3 Groups (#*P* < 0.05 or ##*P* < 0.01), with the most pronounced decline observed in the Z-H3 Group. The hepatotoxicity induced by Zhicaowu in rats was mitigated following the administration of Zhicaowu–Hezi. Among the formulations tested, Zhicaowu–Hezi (1:3) demonstrated the most pronounced efficacy in hepatoprotective effect.

### 3.5 Effect of Zhicaowu–Hezi pairing on mRNA expression of CYP450 enzymes

**FIGURE 9:**
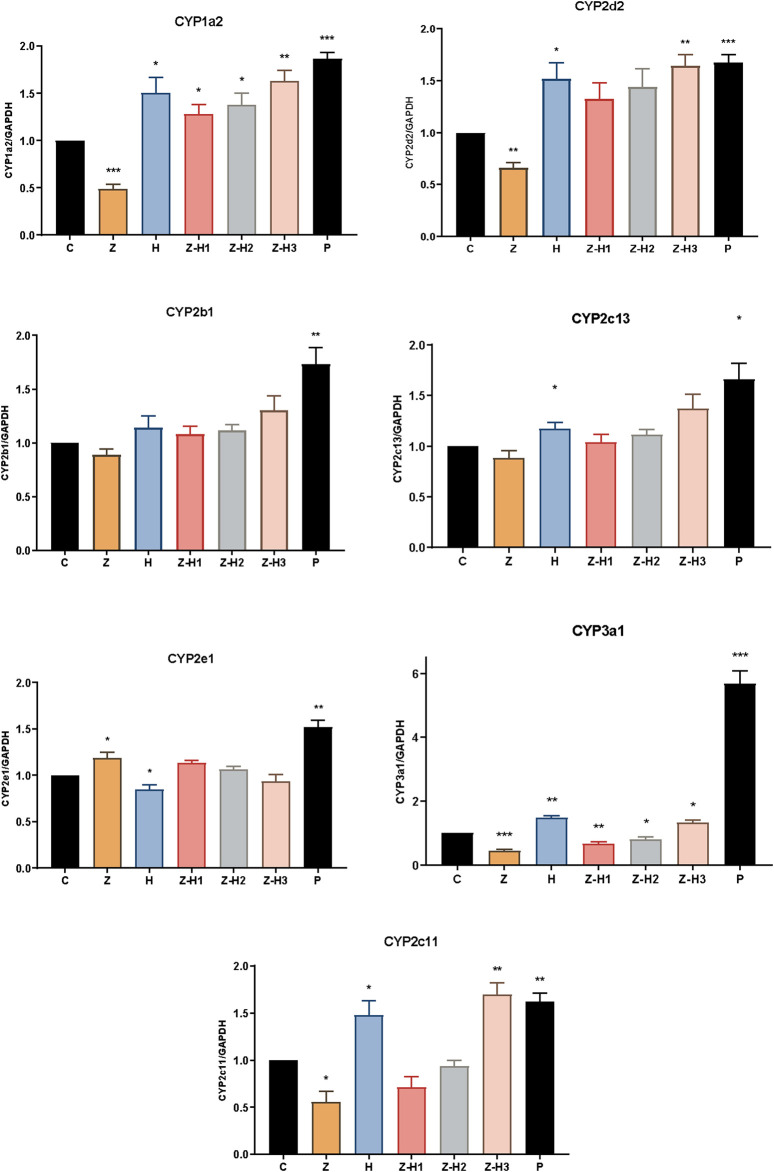
The mRNA expression levels of CYP1a2, CYP2d2, CYP2b1, CYP2c13, CYP2e1, CYP3a1, and CYP2c11 in different groups. Data are expressed as means ± SD. (n = 3). Comparing medication groups with the group C, *P < 0.05, **P < 0.01,***P < 0.001.

mRNA expression levels of CYP1a2, CYP2d2, CYP2b1, CYP2c13, CYP2e1, CYP3a1, and CYP2c11 were subsequently analyzed by RT-qPCR (Figure 9). Compared with Group C, Group Z showed significantly lower mRNA expression levels of CYP1a2, CYP2d2, CYP3a1, and CYP2c11 (*P < 0.05, **P < 0.01, or***P < 0.001). In contrast, the mRNA expression levels of CYP1a2, CYP2d2, CYP3a1, and CYP2c11 significantly increased in the H, Z-H1, Z-H2, and Z-H3 Groups (*P < 0.05 or **P < 0.01). These findings indicate that Zhicaowu downregulated the mRNA expression of CYP1a2, CYP2d2, CYP3a1, and CYP2c11, whereas Zhicaowu-Hezi combination upregulated them. These findings are consistent with both *in vivo* and RLM results.

## 4 Discussion

In recent years, there has been a growing interest among researchers in exploring detoxification mechanisms mediated by metabolizing enzymes. For instance, psoralidin and neobavaisoflavone exert considerable influence on CYP3A4 mRNA expression, expediting drug metabolism and circumventing potential adverse reactions (Shi et al., 2020). Both the Aconitum carmichaelii Debx (Chuanwu) and Caowu belong to the genus Aconite of the Ranunculaceae family. They both contain aconitine-like alkaloids and exhibit therapeutic effects in dispelling wind-dampness, warming meridians and relieving pain. Ge et al. partially elucidated the incompatibility mechanism between Chuanwu and Pinellia ternata (Banxia) - a classic ‘Eighteen Incompatible Medicaments’ pair - via pharmacokinetic analysis and a CYP450 cocktail approach. Their study demonstrated that coadministration of Banxia altered the activity of CYP enzymes (particularly CYP1A2, CYP2D6, CYP3A4 and CYP2C9), thereforemodulating the metabolism of toxic compounds in Chuanwu (Ge et al., 2024). Jin et al. demonstrated that glycyrrhizin upregulated the expression of both CYP3A1/2 and P-gp. Their study found that glycyrrhizin administration (both oral gavage and intravenous injection) significantly reduced the systemic exposure to aconitine by 27% and 33%, respectively. The physiologically based pharmacokinetic (PBPK) modeling for drug-drug interaction (DDI) showed good agreement between predicted and observed data. These results suggest that upregulation of CYP3A1/2 and P-gp serves as a crucial mechanism underlying the detoxification effect between aconitine and glycyrrhizin (Jin et al., 2024). Our pharmacokinetic studies revealed distinct modulation patterns of CYP enzymes between Zhicaowu and Hezi treatments. In rats following a 14-day oral administration, the Zhicaowu group demonstrated significant inhibition of CYP isoforms (rat CYP1a2, CYP2d2, CYP3a1, and CYP2c11), while the Hezi-alone and Zhicaowu-Hezi combination groups exhibited opposite effects, indicating that Hezi induces these enzymes and effectively counteracts Zhicaowu’s inhibitory activity. Notably, no significant alterations were observed in the pharmacokinetics of omeprazole (CYP2c13), bupropion (CYP2b1), or chlorzoxazone (CYP2e1) across treatment groups, suggesting selective modulation of specific CYP pathways.

In the liver microsomal assay, the Zhicaowu group exhibited significantly decreased metabolic clearance rates of theophylline (CYP1a2 substrate), metoprolol (CYP2d2), testosterone (CYP3a1), and diclofenac (CYP2c11), demonstrating Zhicaowu’s inhibitory effect on these CYP450 enzymes. Notably, co-administration with Hezi reversed this inhibition in a ratio-dependent manner. All Zhicaowu-Hezi combination groups showed increased metabolic clearance rates of these substrates, with the most pronounced induction observed in the 1:3 ratio group (Z-H3).

To further validate these findings, RT-qPCR analysis showed that the Zhicaowu-Hezi combination had no significant effect on CYP2b1, CYP2c13, or CYP2e1 mRNA expression, whereas Zhicaowu alone downregulated CYP1a2, CYP2d2, CYP3a1, and CYP2c11, and Hezi alone upregulated these enzymes. Notably, the Zhicaowu-Hezi combination not only reversed Zhicaowu’s suppression but significantly enhanced CYP1a2, CYP2d2, CYP3a1, and CYP2c11 expression above baseline levels, demonstrating Hezi’s transcriptional antagonism of Zhicaowu’s inhibitory effects and providing mechanistic support for the observed pharmacokinetic changes.

Comprehensive analyses using *in vitro*, *in vivo* models, and RT-qPCR revealed that Zhicaowu downregulated the mRNA expression of CYP1a2, CYP2d2, CYP3a1 and CYP2c11, thereby reducing enzyme activity. When combined with Hezi, Zhicaowu’s inhibitory effect was not only reversed but also significantly induced CYP450 expression, with induction being dose-dependent on the content of Hezi. This induction of CYP450 is closely related to the liver protective effect: (1) Normalization of elevated serum AST, ALT and ALP induced by Zhicaowu; (2) Complete prevention of histopathological damage (reducing necrosis and inflammatory infiltration scores to control levels). The metabolic significance of these findings is particularly noteworthy given that aconitine alkaloids - the primary toxic components of Zhicaowu - are predominantly metabolized by CYP3A4 (Ni et al., 2022), with secondary contributions from CYP1A2, CYP2D6, and CYP2C9/19. CYP3A4 is highly expressed in the liver and intestine, which is the most abundant subtype in CYP450 family and participates in about 60% of drug metabolism (Ingelman-Sundberg, 2004). When drugs are co-administered with CYP3A inhibitors or inducers in clinical practice, such combinations may affect their metabolism, slow down/accelerate metabolic rates, increase/decrease their levels, and increase/decrease their toxicity *in vivo*. Hezi exhibits compatibility-modulating and detoxifying effects in herbal formulations. Our data demonstrate that Hezi significantly induces CYP3A4 activity, which likely accelerates aconitine metabolism, thereby reducing systemic exposure and consequent toxicity. This provides a plausible pharmacokinetic mechanism for Hezi’s traditional use as a detoxifying agent in herbal formulations. Building upon these findings, future investigations will focus on two key aspects: (1) quantitative assessment of dynamic changes in toxic alkaloid concentrations following CYP450 enzyme induction, and (2) deciphering the molecular mechanisms of CYP450 regulation in the Zhicaowu-Hezi herb pair.

In conclusion, our *in vivo* and *in vitro* experiments demonstrate that: (1) Zhicaowu significantly suppresses both mRNA expression and enzymatic activity of CYP1a2, CYP2d2, CYP3a1, and CYP2c11; (2) Hezi dose-dependently reverses this inhibition and induces these CYP450 isoforms; (3) The herb combination markedly improves liver function parameters (reduction in ALT/AST) and histopathological outcomes. These findings reveal a potential herb-herb interaction mechanism mediated by CYP450 regulation.

## Data Availability

The original contributions presented in the study are included in the article/Supplementary Material, further inquiries can be directed to the corresponding authors.

## Abbreviations

CYP450, Cytochrome P450; HPLC, High-performance liquid chromatography; RLM, Rat liver microsomes; CMC, Carboxymethyl cellulose; RT-qPCR, Real-time fluorescence quantitative PCR; ALT, Alanine aminotransferase; AST, Aspartate aminotransferase; ALP, Alkaline phosphatase; Group C, Blank control group; Group Z, Zhicaowu group; Group H, Hezi group; Group Z-H1, Zhicaowu–Hezi 3:1 group; Group Z-H2, Zhicaowu–Hezi 1:1 group; Group Z-H3, Zhicaowu–Hezi 1:3 group; Group P, Phenobarbital sodium inducer group; AUC, The area under the plasma concentration-time curve; t1/2 z, Half-life time; CLz/F, Plasma clearance; Cmax, Maximum plasma concentration.
