# Supplementary material for: Potential induction of the relative mRNA expression levels of CYP450 by Zhicaowu-Hezi (Aconiti kusnezoffii radix preparata and Terminalia chebula Retz.)
Source: Front Pharmacol. 2025 Jul 14;16:1573739. doi: 10.3389/fphar.2025.1573739 (PMC12301304; doi:10.3389/fphar.2025.1573739)
Supplement: Supplementary file 1 [file Table1.docx]

TABLE S1

Forward and reverse primers for rat liver metabolising enzyme target and internal reference genes

| CYP450 | Forward primer sequence | Reverse primer sequence |
| --- | --- | --- |
| CYP1a2  CYP2b1  CYP2c11  CYP2c13  CYP2e1  CYP2d2  CYP3a1  GAPDH | CGACAAGACCCTGAGTGAGAAG  GGTGGAGGAACTGCGGAAATC  GAGGACCATTGAGGACCGTATTC  TGTGGTCTTGTTGCTCAGTCTG  TTCTGCTCCTGTCTGCTATTCTG  CCACGAGGAGATTGATGAGGTC  TGCCATCACGGACACAGAAATG  ACGGCAAGTTCAACGGCACAG | GAGGATGGCTAAGAAGAGGAAGAC  AGGAACTGGCGGTCTGTGTAG  GAGCACAGCCCAGGATAAAGG  GGATTGCCGAATGTCCTTCATATC  GATACTGCCAAAGCCAACTGTG  CAGCATTGGTGAACGGCATTC  CTATATCTCTTCCACTCCTCATCCTTAG  CGACATACTCAGCACCAGCATCAC |

TABLE S2

Regression equation and correlation coefficient for seven probe drugs (n = 3)(y = peak area ratio of probe drugs vs IS; x = concentration of probe drugs)

| Probe drugs | Regression equation | Correlation coefficient | Linear range（μg/ml） |
| --- | --- | --- | --- |
| theophylline | y = 0.0273x + 0.0036 | 0.9992 | 0.04—4.05 |
| metoprolol tartrate | y = 0.0597x + 0.0029 | 0.9996 | 0.03—3.30 |
| omeprazole | y = 0.0543x + 0.023 | 0.9979 | 0.05—4.73 |
| bupropion hydrochloride | y = 0.0383x + 0.0092 | 0.9988 | 0.03—2.93 |
| cloxazone | y = 0.1249x + 0.172 | 0.9994 | 0.12—12.23 |
| testosterone | y = 0.1366x + 0.0004 | 0.9998 | 0.03—3.00 |
| diclofenac | y = 0.154x + 0.004 | 0.9988 | 0.05—4.73 |

TABLE S3

Standard curves of probe drugs in rat liver microsomes (RLM) (n = 3)

| Probe drug | Standard curve equation | R^2^ | Linear range（μg/mL） |
| --- | --- | --- | --- |
| Theophylline  Metoprolol  Bupropion  Mephenytoin  Chlorzoxazone  Testosterone  Diclofenac | y = 0.029x - 0.0003  y = 0.0605x - 0.0057  y = 0.0439x - 0.001  y = 0.0425x - 0.004  y = 0.1134x + 0.0095  y = 0.1423x - 0.0141  y = 0.1629x - 0.0046 | 0.9991  0.9979  0.999  0.9994  0.998  0.999  0.9983 | 0.08—0.75  0.80—8.00  0.46—4.60  0.91—9.10  0.29—2.85  1.20—12.00  0.27—2.65 |
